# Virus Infection Is an Instigator of Intestinal Dysbiosis Leading to Type 1 Diabetes

**DOI:** 10.3389/fimmu.2021.751337

**Published:** 2021-10-15

**Authors:** Zachary J. Morse, Marc S. Horwitz

**Affiliations:** Department of Microbiology and Immunology, University of British Columbia, Vancouver, BC, Canada

**Keywords:** type 1 diabetes, microbiome, autoimmunity, coxsackievirus, dysbiosis, gut

## Abstract

In addition to genetic predisposition, environmental determinants contribute to a complex etiology leading to onset of type 1 diabetes (T1D). Multiple studies have established the gut as an important site for immune modulation that can directly impact development of autoreactive cell populations against pancreatic self-antigens. Significant efforts have been made to unravel how changes in the microbiome function as a contributor to autoimmune responses and can serve as a biomarker for diabetes development. Large-scale longitudinal studies reveal that common environmental exposures precede diabetes pathology. Virus infections, particularly those associated with the gut, have been prominently identified as risk factors for T1D development. Evidence suggests recent-onset T1D patients experience pre-existing subclinical enteropathy and dysbiosis leading up to development of diabetes. The start of these dysbiotic events coincide with detection of virus infections. Thus viral infection may be a contributing driver for microbiome dysbiosis and disruption of intestinal homeostasis prior to T1D onset. Ultimately, understanding the cross-talk between viral infection, the microbiome, and the immune system is key for the development of preventative measures against T1D.

## Introduction

Type 1 diabetes (T1D) is a persistent autoimmune disorder where immune cells attack and destroy the insulin-producing beta cells of the pancreas. Eventually, once enough beta cell mass is lost, individuals will begin to experience loss of natural blood glucose regulation and become reliant on exogenous administration of insulin. Numerous studies have characterized genetic variance and single nucleotide polymorphisms associated with T1D, which can explain why some individuals are more predisposed than others (1–6). Genome-wide association studies have found that Human leukocyte antigen (HLA) and insulin genes are responsible for a significant portion of the genetic risk for T1D. Additionally, many polymorphisms have been identified within immune-related genes including *PTPN22*, *IFIH1*, *CTLA4*, and *IL2RA* (5, 6). However, genetic make-up only accounts for part of the equation. After all, the immune system is shaped to an incredible extent by non-heritable forces and instead moulded largely by environmental exposures (7).

An array of exogenous stressors have been associated with precipitating autoimmunity (8). However, understanding exactly how environmental factors contribute to disease pathogenesis is a messy ordeal. Dysbiosis, infection, exposure to dietary antigen, and vitamin D deficiency have all been significantly implicated in altering susceptibility to T1D (9, 10). With such complicated etiology, incorporation of multi-faceted approaches, which take into account the extensive amount of cross-talk that occurs between each of these influences on the host, should be strongly considered in future studies.

**Figure 1:**
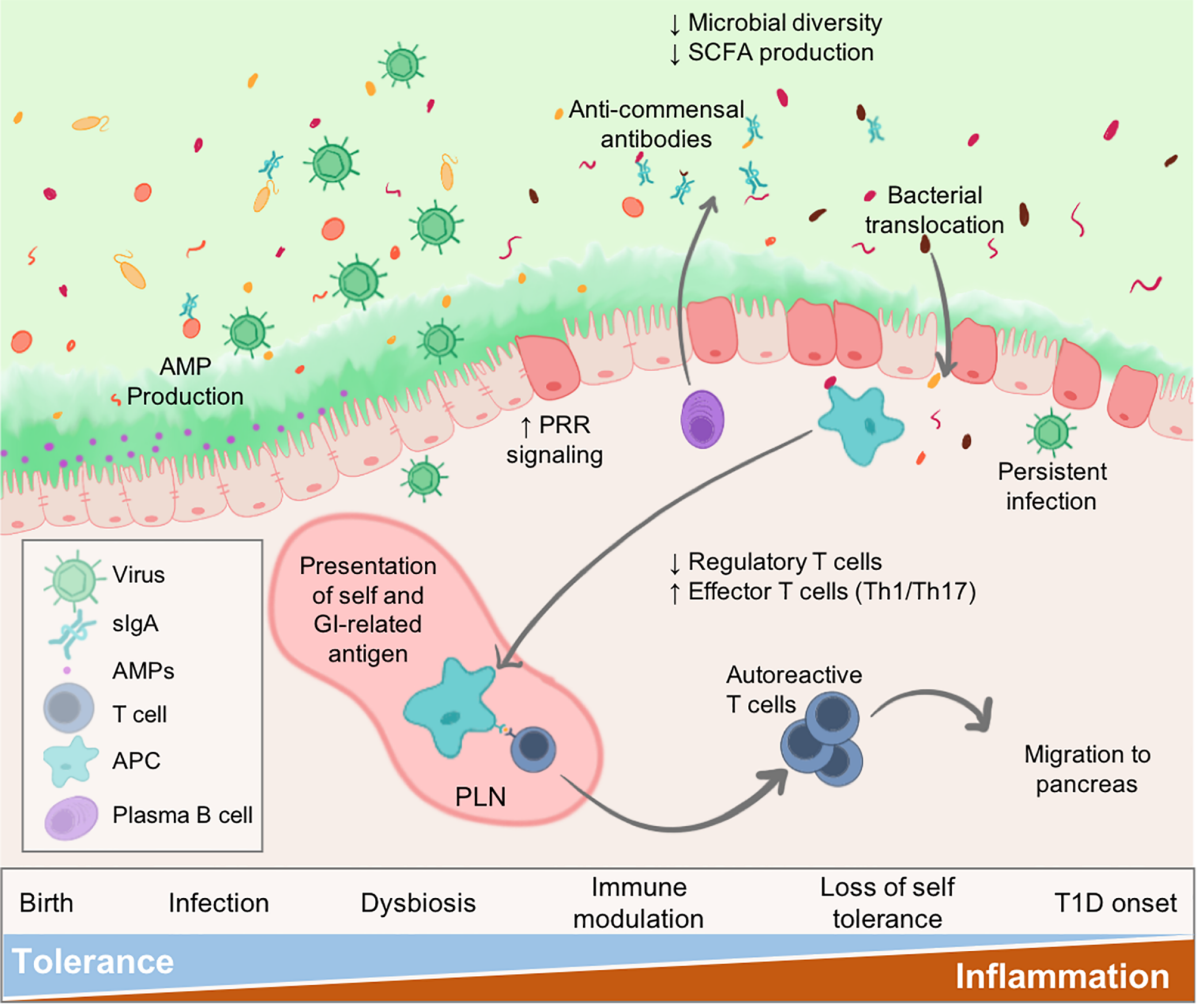
Virus infections alter intestinal homeostasis to contribute to T1D. The GI environment is tightly regulated by numerous mechanisms. Perturbations such as virus infection results in dysbiosis and disruption to the enteric environment. Microbial dysbiosis is characterized by loss of species diversity and production of SCFAs including butyrate and acetate. As a result of dysbiosis and inflammation, the epithelial barrier becomes more permeable due to loss of tight junctions between epithelial cells, alteration of secreted IgA (sIgA) antibodies, and diminished mucus production. Some persistent infections may be maintained contributing to sustained inflammatory signalling within the gut. Both pancreatic self-antigens and commensal microbial antigens are taken up by APCs and presented to T cells in the pLN causing loss of self-tolerance. These autoreactive T cells migrate to the pancreas to contribute to anti-islet responses and destruction of insulin-secreting beta cells. Individuals would progress to T1D once sufficient beta cell mass is lost resulting in loss of blood glucose regulation.

Virus infections may be an instigating factor for the gut pathology and dysbiosis that is observed in patients leading up to islet autoimmunity and/or T1D onset (**Figure 1**). Clinical evidence suggests that diabetic patients experience prolonged enterovirus infections associated with the gut mucosa, resulting in persistent inflammation. Furthermore, patients with islet autoimmunity have increased intestinal permeability, low-grade enteropathy, and a dysbiotic microbiome. Seasonal patterns observed in T1D and other autoimmune disease diagnosis could, at least partially, be explained by seasonal variations in infection (11, 12). In this review, we will examine the known effects of virus infection on the microbiome and gastrointestinal (GI) physiology, and how this modulation may relate to T1D pathogenesis.

## Virus Infections Are Associated With T1D

Numerous viruses, particularly those associated with the gut, have been connected with T1D pathogenesis including enterovirus, rotavirus, cytomegalovirus, and norovirus (13–17). The enterovirus, coxsackievirus B (CVB), has been the virus most frequently associated with T1D. So much so, that recently there has been movement and discussion towards the necessity to develop a vaccine specific for coxsackievirus to help mitigate the globally increasing rates of T1D (18–21). CVB binds to the coxsackie and adenovirus receptor (CAR), which is highly expressed on the insulin-secreting beta cells in the pancreatic islets (22). Variance in CAR expression has been correlated with increased predisposition for T1D (23). In both human populations and experimental mouse models, infection with enteroviruses has been identified to precede onset of islet autoimmunity (24–26). A recent large-scale study looking at virus shedding in the stool of children found that while those with islet-autoantibodies had no difference in total incidence of infection, they did experience a higher rate of sustained enterovirus B (particularly of CVB serotype) infections, which may be contributing to islet autoimmunity (23). Interestingly, this study also found association of other mammalian viruses including human masadenovirus-C (HAdV-C), which actually correlates with reduced incidence of auto-reactivity. The authors suggest that this may be due to HAdV-C competitively inhibiting CAR engagement or through sustained activation of innate immunity resulting in protection from other strains of virus including enterovirus. Children who developed T1D and islet-specific autoantibodies also have a history of increased incidence of respiratory infections in early adolescence (27). It is unclear, however, if there may be underlying immune differences that cause these populations to have increased susceptibility to both these types of infections and T1D autoimmunity. But, children who experience early loss of B cell tolerance to insulin exhibit weak humoral protection against CVB, whereas those with autoantibodies to the T1D biomarker, glutamic acid decarboxylase (GAD), have competent CVB responses - signifying viral clearance may be altered in individuals with T1D-related autoimmunity (28).

Rotavirus infection in children with a genetic predisposition to T1D is associated with increased islet autoimmunity, signifying that infection may exacerbate autoimmunity and diabetes (14). In non-obese diabetic (NOD) mice, rotavirus infection has also been shown to accelerate onset of T1D (29). However, pre-existing autoimmunity is necessary to accelerate disease onset (29). Thus, rotavirus may likely promote pathogenic events rather than serving as a trigger of diabetes.

Antiviral responses to viruses including CVB can likely have direct effects within the pancreas in precipitating T1D (30–32). While CVB has been shown to impair beta cell function *in vitro*, evidence suggests that the virus itself does not destroy beta cells through cytopathy (30, 33). Antiviral responses are largely mediated through expression of the three classes of interferon (IFN): type I (IFN-α and IFN-β), type II (IFN-γ) and type III (IFN-λ). Innate viral receptor engagement and ensuing immune pathway activation can have a significant role in T1D initiation and pathogenesis (34, 35). A transient type I IFN signature has been observed preceding islet autoantibody development in genetically-susceptible children, but is lost by the time of diabetes diagnosis (36–38). This IFN signalling may be a significant contributor to the hyperexpression of major histocompatibility complex (MHC) class I, endoplasmic reticulum stress, epigenetic and transcriptional/translational modifications observed in the islet microenvironment prior to T1D development. Recently, researchers were able to detect viral signatures (enteroviral protein and dsRNA) in the islets of autoantibody-positive and recent-onset T1D donors along with increased interferon and microbial stress markers (39). There has also been some suggestion that terminally-deleted viral genomes are able to persist in the islet microenvironment causing inflammation and increased immune cell recruitment (40). While there are existing direct links to virus-causing inflammation and modulation of the immune system within the islet microenvironment, there are likely secondary effects of infection, which are also long-term contributors to disease pathogenesis including microbial dysbiosis.

## The Microbiome and T1D

Comprising of a rich diversity of bacteria, archaea, viruses, fungi, and helminths – the microbiome is a dynamic environment that is constantly shifting. This review primarily focuses on the impact of commensal bacterial communities and later the collective virome. The microbiome has a substantial role in shaping peripheral immune tolerance, activation, migration, and differentiation, as well as local inflammatory responses (41). In response, the immune system is in constant communication to respond to these fluctuations in stimuli (42). Alterations in the microbiome have been heavily implicated in the pathogenesis of T1D (43–45) and genetic risk for T1D autoimmunity even confers differences in the bacterial microbiome (46–48). The intestinal microbiota can exert potent influence on immune homeostasis through the production of various metabolites and particularly short chain fatty acids (SCFAs). Both clinical studies and mouse models have established SCFAs including butyrate, propionate, and acetate as significant factors affecting immune regulation in T1D pathogenesis (43, 49). Metabolite-related dietary patterns have been shown to influence T1D susceptibility and metabolomic alterations precede the development of islet autoantibodies in children (50, 51).

While the human microbiome can be quite heterogenous and studies examining the relationship between the microbiome and diabetes have produced highly variable results, there are some notable microbial hallmarks which have been often identified in individuals with T1D and islet-autoimmunity including: a marked decrease in the diversity of bacteria colonizing the gut, increased abundance of bacteria within the *Bacteroides* phylum, the loss of *Firmicutes*, and decreased production of SCFAs among other variances (52–55). However, understanding the effects of perturbations in humans while also controlling for extraneous factors is incredibly difficult. The use of NOD mice as a model for spontaneously developing diabetes has given significant insight into understanding the disease pathogenesis of T1D. While the autoimmunity experienced by NOD mice is not the same as that experienced by humans, it allows the use of environmental and genetic interventions in order to understand how they may impact diabetes development (56). Dysbiosis occurs in both humans and NOD mice prior to disease onset and diabetes incidence can even be predicted in these mice based on sampling from various mucosal microbiomes (43, 57, 58). A “diabetogenic” microbiome from a diabetes-prone NOD mouse is sufficient to promote insulitis when transferred to a non-obese diabetes-resistant (NOR) mouse (59). Typically, female NOD mice are more susceptible to developing autoimmune diabetes than male mice; however, this difference is not observed in germ-free mice (60). This discrepancy in sex bias can at least partially be explained by microbial stimulation of testosterone (60).

Pattern recognition receptors (PRRs) including toll-like receptors (TLRs), RIG-I-like receptors, and caspase activation and recruitment domains (CARDs) are innate sensors that can detect viral and microbial pathogen-associated molecular patterns (PAMPs). Signalling through these receptors can be detrimental for modifying susceptibility to T1D development (34). There are 13 total types of TLRs, each of which is specific for various bacterial (LPS, flagellin, peptidoglycan, etc.) and/or viral (dsRNA, CpG DNA, viral protein, etc.) antigens. Microbiota can regulate T1D through TLR signaling differences (61, 62). For instance, imbalance between TLR2 *vs*. TLR4 stimulation can determine T1D susceptibility where TLR2 provides a pro-diabetic signal whereas TLR4 provides microbiota-induced tolerization (61). This overlap in bacterial and virus infection immune signaling may signify a role between commensal microbes and virus infection in host immune regulation.

## Virus Infections as a Source of Dysbiotic Perturbation

In the first few years of life, colonization of the GI tract plays an indispensable role in shaping host immune development, regulation, and maintenance (63, 64). With age, the microbiome experiences decreasing plasticity and tolerance for new antigen exposure and environmental disruptions (63, 65). Following infancy, the microbiome seems to stabilize with relatively established communities that continue to shape mucosal and systemic immune homeostasis into adulthood (66). Thus, timing of environmental perturbations is likely an important factor for producing dysbiosis, which impacts disease susceptibility. The “Hygiene Hypothesis” proposes that exposure to antigens in early life during immune development can have profound effects for the development of autoimmune and allergic disease later on. Evidence suggests that instigating factors leading to T1D occur early in life – especially since a majority of early-onset individuals who progress to overt T1D before adolescence develop autoantibodies by 3 years of age (1, 67). However, most individuals are diagnosed with T1D in adulthood, hinting that tolerance for environmental stressors may not necessarily be limited to a defined age or that triggering events can occur long before disease onset (68).

Infections that are relatively mild later in life, may have the ability to be quite detrimental early in life at promoting T1D, as the immune system is not yet fully developed and may lack the ability to properly defend the host (69). Viruses cause dysbiosis (70–72), potentially signifying lasting consequences whereby individuals may develop a more autoimmune-skewed microbiome that might be characterized by decreased diversity and less beneficial bacteria (e.g., less butyrate producers). Studies in NOD mice have shown early life exposure to a “diabetogenic microbiome” through fecal microbiome transfers (FMT) can regulate B cell activation and promote T1D onset later on (69). However, when mice are given this same microbiome composition post-adolescence they do not experience the same modulation of the immune system and increased incidence of diabetes autoimmunity. Thus, there may be a unique window, particularly early in life, whereby disruptions in the microbiome from exogenous stressors like infection can have much larger implications on future health.

## T1D: A Consequence of Intestinal Dysbiosis and Resident Immune Population Control

Studies have found that detection of enteric infection precedes islet autoimmunity by 6 months or more (24, 73, 74). The existing confluence between intestinal inflammation and T1D maintain the gut as an important site for immune modulation that has implications for islet autoreactivity. While some viruses may have deleterious effects on the microbiome, others may actually promote tolerance. For example, norovirus infection was shown to protect from T1D through modulation of the microbiome (75). In this study, Pearson et al. found that NOD mice infected with murine norovirus (MNV) had significantly lower diabetes incidence, less immune infiltration into the islets, increased bacterial diversity, and an increased regulatory rather than inflammatory T cell profile.

Islet-autoreactive CD8^+^ T cells circulate in the blood in approximately the same quantities between healthy and diabetic patients – suggesting that these cells are a normal part of the T cell repertoire (76). However these cells are more abundant in the pancreata of T1D patients, indicating that they must home to the pancreas due to altered immunoregulatory signalling, proinflammatory islet environment, and/or peripheral activation (76). The GI tract plays a fundamental part in communicating between the host and microbiota. Even at healthy steady-state conditions, there is significant T cell trafficking between the gut and pancreatic tissues (77). Existing within this gut-pancreas axis, the pancreatic lymph nodes (pLN), which drain from the duodenum and pancreas, are sampling antigen heavily from both organs. The pLN resides at a critical and significant confluence whereby intestinal stress can alter the presentation of pancreatic self-antigens (77). It has even been suggested that this may be the portal connecting celiac disease with T1D, where GI inflammation due to gluten-sensitivity potentially stimulates anti-islet immune activation in the pancreas (77, 78). Diabetic patients experience prolonged enterovirus infections associated with the gut mucosa, resulting in persistent inflammation (79). This sustained inflammation may be sufficient to result in loss of self-tolerance and T1D development.

### Adaptive Cells

Resident T and B cells hold specificity for commensal microbes even under healthy homeostatic conditions (80). T cell polarization into T helper 1 (Th1), Th2, Th17, or regulatory (Treg) cell phenotypes can be driven in the gut by presence and abundance of specific microbes in autoimmunity (42, 81, 82). For instance, *Bifidobacteria* species can drive Th17 cell responses (83) – while *Akkermansia*, *Bacteroides*, and most notably *Clostridium* species, have been shown to promote Treg populations (82, 84). Produced in large quantities, particularly by *Clostridium* bacteria, the SCFA butyrate is a potent inducer of Treg delineation through histone modification promoting *Foxp3* expression and, by eliciting high levels of transforming growth factor β (TGF-β), expression in gut-related CD103^+^ dendritic cells (85, 86).

### Regulatory T Cells

Insight into the pathogenesis of T1D has revealed that Treg cells can be potent mediators for the suppression of autoreactive T cells and promotion of tolerance to islet antigen (87). Inflammasome-deficient mice have a microbiome that is protective for T1D (88). When NOD mice are co-housed with these protected mice they experience a corresponding reduction in diabetes incidence (89). This is attributed to an expansion of type 1 regulatory T cells in the gut, which home to the pancreas and secrete IL-10 to reduce inflammation in the pancreatic microenvironment. This microbiome-driven alteration in Treg populations is likely due to production of bacterial SCFA metabolites since administration of butyrate to NOD mice also causes initial expansion of Tregs in the colon, mesenteric lymph nodes (mLN), and Peyer’s patches with a subsequent migration to the pancreas and pLN to reduce T1D onset (90). Expansion of *Ruminococcus* species of bacteria can also promote CD8^+^ Treg cells to prevent diabetes in NOD mice and a streptozotocin-induced model. Furthermore, healthy human donors have increased CD8^+^ Tregs along with increased *Ruminococcus* when compared to T1D patients (91). These gut-primed Tregs may have a profound impact on maintaining pancreatic tolerance and may be limited in infection since enterovirus detection in young children is associated with ensuing depression of Treg responses and increased inflammatory Th1/Th17 responses (92).

### Mucosa-Associated Invariant T Cells

Mucosa-associated invariant T cells (MAIT) are innate-like T cells expressing MHC class 1-related protein (MR1) that specifically binds microbial metabolites originating from riboflavin metabolite biosynthesis in bacteria. These cells are present in several tissues, and like their name suggests, they are important at mucosal sites (93). MAIT cells exist at an interesting interface and may be a key mediator between microbes, virus infection, and T1D. Germ-free mice lack MAIT cells, thus indicating that they likely rely on commensal bacteria for their development and maintenance (93). In fact, differences in bacterial metabolism can regulate MAIT cell activation (94, 95). Typically, MAIT cells are thought to have a protective phenotype whereby they promote intestinal homeostasis and have a role in supporting the gut epithelial barrier *via* secretion of IL-22, IL-12, and IL-17a (96). However, MAIT cells can also take on a more pathogenic nature in certain circumstances.

Rouxel et al. found that both recent-onset and established T1D patients have altered MAIT cell populations circulating in their blood whereby they are less abundant, express more activation/exhaustion markers, Th1-skewed, and are more cytotoxic (97). In NOD mice, MAIT cells seem to show a dimorphic phenotype depending on tissue specificity where MAIT cells in the lamina propria express IL-22 and IL-17a in non-diabetic mice; however, cells that infiltrate the pancreatic islets express IFN-γ and granzyme B to participate in beta cell destruction. Furthermore, the authors showed that MAIT cell-deficient (MR1-restricted) NOD mice have increased rates of diabetes and have a modified gut mucosal environment – suggesting that they can be protective (97). Beyond their role in sensing bacterial products, MAIT cells have also been identified to hold potent inflammatory responses in both acute and chronic virus infections (98). This is due to activation, which is independent of MR1 stimulation and instead due to cytokine signaling largely through type-1 interferon, IL-12, and IL-18 (98, 99). Ultimately, collective signalling from bacterial metabolites and cytokine profiles in infection may be detrimental in skewing MAIT populations toward either a protective or pathogenic nature in T1D pathogenesis.

### B Cells

Mariño et al. found that providing diets to NOD mice that yield increased production of acetate and/or butyrate are largely protected from autoimmune diabetes (49). These two SCFAs accomplish this through their own distinct mechanisms. While butyrate primarily boosted Tregs, acetate decreased frequency of islet-specific autoreactive T cells by modulating antigen presentation in B cell populations residing in the spleen and intestinal Peyer’s patches. Cross presentation of islet antigen by B cells in the pLN has been previously been shown to activate self-reactive CD8^+^ T cells (100).

### Antigen-Presenting Cells

Plasmacytoid dendritic cells (pDC) play an important part in mediating antiviral intestinal immunity. These cells extend their dendrites across the epithelial cell barrier to sample microbial antigen in the GI tract to present to resident adaptive immune cells and can produce a significant amount of IFN in infection. pDCs infected with rotavirus can induce bystander activation of islet-reactive T cells *via* type I interferon signalling (17). Mucosa-associated pDCs likely detect virus infection and travel to the mesenteric and/or pLN to promote B cell expression of MHC-I and proinflammatory T cell cytokine secretion to aid in inflammation (101, 102). Phagocytosis of *Lactococcus lactis* bacteria by pDCs can stimulate robust IFN-α secretion *via* TLR9 and MyD88 signalling (103). Oral administration of the *L. lactis* colonization factor antigen I fimbriae can also prevent T1D in NOD mice by promoting expansion of IL-10 and IFNγ while decreasing Th1 T cells (104).

MNV infection alters recruitment of macrophages in the pLN where they are deficient in CD86, signifying a decreased capacity to activate T cells leading to protection from T1D (75). Furthermore the offspring of antibiotic-treated pregnant NOD mice also experience reduced T1D incidence by instigating tolerized APCs (105). These APCs have a diminished ability to activate cytotoxic CD8^+^ T cells and thus represent the importance for microbiome-specific education of developing immune self-tolerance. Macrophages which lack previous exposure to bacteria in antibiotic-treated mice have reduced responses to LPS antigens (106). Decreased inflammatory responses by these APC populations due to microbiome differences may be sufficient to prevent autoreactivity – especially since islet resident macrophages are detrimental for the instigation of T1D autoimmunity in NOD mice (107).

## Infection and a Leaky Gut

Containment of commensal bacteria and dietary antigens within the intestinal lumen relies on several physiological and molecular barriers. The first line of defense is a layer of mucus created by O-linked glycoproteins (mucins) secreted from intestinal goblet cells combined with luminal saccharides. In the colon, a double layer of mucus serves as a physical barrier. The apical layer is typically colonized with various mucus-degrading microbes including those within the *Akkermansia* family. The innermost mucus layer, however, is predominately uncolonized and creates a largely impenetrable barrier for bacteria. A single cell layer of epithelial cells (IEB) is joined through tight junctions to create a continuous cellular barrier throughout the GI tract. This IEB can be maintained by cytokines including IL-22 produced by group 3 innate lymphoid cells and IL-17A from Th17 lamina propria T cells. Epithelial cells and resident lamina propria immune cells constantly sample the mucosal environment and respond to changes in microbial and viral stimuli. Commensal bacteria populations are regulated through production of antimicrobial peptides (AMPs) and by secreted IgA antibodies. AMPs are bactericidal for specific bugs, particularly within the small intestine where the mucus barrier can be more discontinuous. Colonization of bacteria within the GI tract is also highly regulated by IgA antibodies, which can coat bacteria for neutralization and opsonization. Most secreted IgA is polyreactive and holds an innate specificity to multiple strains of bacteria, but can also undergo somatic hypermutation to produce highly specific IgA against particular bacteria (91, 108).

Autoimmune disorders including T1D, rheumatoid arthritis, multiple sclerosis, and systemic lupus erythematosus (SLE) have all been associated with increases in intestinal permeability - or a so-called “leaky gut” (109–111). Clinical studies have found that individuals with islet autoimmunity experience increased intestinal permeability and low-grade enteropathy (112–116). Loss of integrity occurs prior to T1D development in both human and mouse models, indicating that it may be a significant trigger – rather than a result – of autoimmunity (112, 117). In fact, Sorini et al. found that breaking the intestinal barrier using low-dose dextran-sulfate sodium (DSS) treatments in NOD mice was sufficient to increase onset of autoimmune diabetes (109). This subsequent loss of intestinal integrity can induce activation of islet-specific immune cells in the gut to travel to the pancreas and promote onset of diabetes in T cell receptor-transgenic BDC2.5 crossed NOD mice. Activation of these T cells also appeared to be dependent on the presence of the gut microbiome; however, microbial dysbiosis caused by the DSS-treatment alone was not sufficient to promote autoimmunity.

### Bacterial Translocation

Breakage of the tight junctions, which glue together the intestinal epithelial barrier, may be a contributing factor in allowing permeability and contribute to T1D pathogenesis (118). As a result of reduced intestinal integrity, bacteria can cross mucosal barriers and leak into systemic circulation and various tissues. When disseminated systemically, commensal bacteria antigens can rapidly promote diabetes autoimmunity in NOD mice (119). Translocation of bacteria can contribute to autoreactivity in the following ways: 1) by directly damaging the beta cells (120) 2) through presentation of bacterial antigen to autoreactive T cells (109) 3) in promoting inflammation through innate receptor stimulation (121) 4) through bacterial molecular mimicry of self-antigens (122). In fact, translocation to the pLN has been observed in NOD mice prior to diabetes onset (123). It can also trigger activation of the innate bacterial peptidoglycan receptor, NOD2, to contribute to T1D development in a streptozotocin-induced mouse model (121). Islets exposed to translocated bacteria can directly mount anti-bacterial responses and promote inflammation (124). These responses may ultimately aid in recruitment and activation of autoreactive cells within the pancreatic environment.

### Intestinal Homeostasis in the NOD Mouse

Miranda et al. performed an extensive analysis looking at alterations at the mucosal immune environment in NOD mice prior to diabetes development (123). This study found that the mice developed impaired mucin production, dysbiosis, modified secretion of bacteria-specific IgA, and alterations in lamina propria dendritic and T cell populations – which skew toward an inflammatory rather than regulatory profile. Some of these changes were shown to be microbiome-driven since cross-fostering NOD pups with NOR mothers can restore mucus production. The intestinal mucus layers represent the first line of defense against intestinal microbes and can be modulated by the presence of specific bacteria. Specifically, butyrate-producing and mucin-degrading bacteria can improve intestinal integrity through regulation of epithelial tight junctions and stimulate production of mucin synthesis, respectively (49, 125, 126). Acetate- and butyrate-yielding diets correspond to a reduced concentration of bacterial lipopolysaccharide (LPS) antigens detected in the serum of mice - indicating reduced bacterial dissemination (49). The mucin-degrading bacteria, *Akkermansia muciniphilia*, can reduce intestinal permeability through fortification of epithelial tight junctions (126). Administration of *A. muciniphilia* can reduce diabetes incidence in NOD mice by modulating mucus production and expression of antimicrobial peptides (127). Furthermore, the colonization of *A. muciniphilia* decreased islet expression of TLRs and promoted regulatory T cells (127). This potentially signifies a change in the host’s ability to respond to subsequent infections and susceptibility to infection-induced diabetogenic responses.

### Commensal-Specific Antibody Responses

Alterations in the abundance of certain bacterial antigens have been previously observed to elicit specific IgG antibody responses to commensal bacteria suggesting that B cell receptor and TLR stimulation can alter GI-related B cell profiles (128). Furthermore, SCFA metabolite concentration can drive production of bacteria strain-specific IgA in a T cell-dependent mechanism involving TLR recognition – resulting in altered bacterial colonization of mucosal environments (108). The presence of T1D and/or autoimmune risk alleles confers alterations in IgG and IgA anti-commensal microbial responses in HLA haplotype-dependent and -independent mechanisms (129). For example, Huang et al. observed that newly-diagnosed T1D patients have increased secretory IgA responses along with dysbiosis and decreased SCFA production (130). Performing FMTs to transfer the microbiota from these T1D patients to germ-free NOD mice results in similar alteration in IgA-mediated immunity in these mice. However, administration of the SCFA acetate is able to recover this modulation and restore IgA responses. It has yet to be determined if dysregulation of IgA-mediated control of commensal bacteria communities and intestinal homeostasis has role in contributing to T1D autoimmunity or if it is a by-product of dysbiosis and/or metabolic pathogenesis. Some evidence has indicated that changes in the anti-commensal antibody milieu occurs after seroconversion, but prior to T1D onset (48).

### Infection as an Instigator of Intestinal Permeability

Collectively, research included in this review suggests that a “leaky gut” is a natural part of T1D pathophysiology that likely triggers and/or progresses disease (**Table 1**). Virus infections may be a causative agent to aid in microbiome-related promotion of autoreactivity. Increased gut inflammation invariably leads to loss of epithelial integrity and a breakdown of the barriers – thereby allowing dissemination of bacteria from the gut and increasing immune accessibility to antigens within the GI tract. Chronic viral infection is sufficient to drive sustained intestinal permeability (133). This infection-induced epithelial damage can be mitigated through blockade of type I IFN or depletion of CD8^+^ T cells (133). Infection with *Citrobacter rodentium* is able to produce barrier disruption along with increased insulitis in NOD mice (134). Respiratory infections are known to cause gastrointestinal distress, dysbiosis, and increased intestinal permeability despite an absence of virus in the GI environment (135). SARS-CoV2 patients experience noted dysbiosis and loss of intestinal integrity corresponding with more severe systemic inflammation, bacteremia, and higher mortality rate – potentially signifying a leaky gut as a contributor to worsening disease outcomes (136, 137). Additionally, human immunodeficiency virus (HIV) infection has been shown to cause systemic immune activation and AIDs-related morbidity due to translocation of bacteria from the intestinal lumen (138). In fact, HIV positive individuals can experience systemically disseminated bacteria resulting in stimulation of anti-CD4^+^ T cell autoantibody production (139).

**Table 1:** Highlighted recent studies depicting intestinal changes associated with T1D.

Organism	Virus	Result	Microbiome Dysbiosis	Intestinal Pathology	Intestinal Immune Changes	Ref
NOD mice	None	Butyrate and acetate SCFA administration protects from T1D	Increased *Bacteroides*	SCFA treatment reduced systemic bacterial translocation and increased expression of tight junction proteins	SCFA treatment promotes increased Treg populations, altered B cell differentiation and function, increased serum IL-22, and decreased serum IL-21.	Marino et al. (49)
NOD Mice	None	NOD mice receiving FMT from T1D patients had modified IgA immunity to GI bacteria. Acetate treatment reverses IgA dysfunction.	Decreased diversity, decreased *Firmicutes* in mice receiving FMT from T1D patients	NOD mice receiving FMT from T1D donors experience heightened intestinal permeability, increased IgA immunity, and decreased AMP expression	Acetate treatment increases gut-associated Tregs and decreases IgA+ B cells.	Huang et al. (130)
NOD mice	None	Low-grade DSS administration is able to induce T1D.	DSS treatment alters microbiome, however FMT of dysbiotic DSS-induced microbiome to naïve mice is insufficient to promote T1D alone	Increased permeability triggers T1D (NOD mice have decreased tight junction protein expression, and reduced mucosal barrier	Increased intestinal permeability activates islet-reactive T cells and increased gut related T cell infiltration into the pancreatic islets.	Sorini et al. (109)
NOD mice	None	Intestinal homeostasis is altered in NOD prior to T1D onset.	Increased *Firmicutes* and reduced *Actinobacteria* prior to T1D development	Prediabetic NOD mice have increased intestinal permeability, diminished mucus production, bacterial translocation, and reduced IgA.	Prior to T1D onset, mice have elevated Th1 and Th17 responses as well as decreased Th2 cells, ILC2s, and Tregs in the small intestine.	Miranda et al. (123)
NOD mice	None	TLR4-defiecient NOD mice have accelerated T1D onset.	T1D was associated with increased *Bacteroides*, lower *Firmicutes*, and decreased peripheral SCFA levels.	Increased bacterial translocation (Serum LPS levels)	ND	Simon et al. (62)
NOD mice	None	Offspring of NOD mice treated with Vancomycin had increased autoimmunity and those treated with Neomycin experienced protection.	Both case group mice had less segmented filamentous bacteria. Offspring of neomycin-treated mice had less gram-positive bacteria overall, and more *Actinobacteria*.	ND	Neomycin-treated mice had significantly less co-stimulatory molecule expression on APCs, and decreased Th1 and Th17 T cells.	Hu et al. (105)
NOD mice	MNV	MNV infection protects from T1D development.	Increased alpha-diversity, increased *Firmucutes*/*Bacteroides* ratio, and reduced *Akkermansia* in infected mice	MNV infection causes altered Tuft cell gene expression. No changes in permeability, tight junction, or AMP expression in infected mice.	Infected mice had increased systemic Tregs, reduced inflammatory T cells and cytokine secretion, altered mucosa-associated B cell populations, and increased macrophage recruitment in pLN	Pearson et al. (75)
Humans	Unknown	Human T1D patients have decreased acetate levels and increased IgA production.	T1D patients had increased bacterial diversity, with decreased *Firmicutes* species prevalence, and decreased stool acetate and butyrate levels	T1D patients had increased IgA-coated bacteria in their stool.	ND	Huang et al. (130)
Humans	Enterovirus	Small bowel mucosa from T1D patients have increased prevalence of enterovirus. Children who progress to T1D experience sustained enterovirus infections prior to autoimmunity.	ND	Virus positive and T1D patients had increased mucosal IgA deposits.	Virus positive patients had increased CD3 intra-epithelial leukocytes. T1D patients (without celiac disease) had increased HLA-DR expression.	Oikarinen et al. (79) Honkanen et al. (24)
Humans	Unknown	Children with islet autoantibodies and who progress to T1D experience intestinal dysbiosis.	Case subjects had decreased anti-inflammatory Prevotella and Butyricimonas bacteria as well as overall decreased microbial diversity.	Individuals with islet autoantibodies and those who progressed to T1D had increased intestinal permeability and decreased mucus production	Seropositive subjects had decreased IgA (decreased stool IGHA1)	Harbison et al. (115) Gavin et al. (131)
Humans	Enterovirus B and intestinal virome	Children with islet autoantibodies experience sustained enterovirus B shedding. Changes in the virome precede T1D-related autoantibody detection.	Genetic risk for T1D confers altered virome. Increased prevalence of *Bacteroides dorei* bacteria and *Bacteroides*-associated phages prior to seroconversion.	ND	ND	Vehik et al. (23) Zhao et al. (132)

ND, no data.

## Virome as a Contributor to Host Immunity and Microbial Regulation

The intestinal virome is made up of rich and diverse prokaryotic and eukaryotic viral communities, which are shaped by numerous factors including diet, genetics, disease, and geography (140). While a vast majority of the viruses in the body are bacteria-infecting phages, the human virome is also made up of: genomically-integrated human endogenous retroviruses (HERVs); latently-infecting viruses, such as human herpes viruses (HHVs); and potentially persistent/chronic infections – including common enteric viruses previously discussed in this review (CVB, norovirus, rotavirus, etc.) (23, 141). With the GI tract being the most abundant site of viral colonization, the intestinal virome is crucial for maintaining homeostasis and regulating disease pathogenesis through interaction with both commensal bacteria as well as the host (142). Typically germ-free and antibiotic-treated mice face immune dysfunction and altered intestinal morphology. However, infecting these mice with MNV mitigates these aberrations in the intestinal environment (143). Norovirus is therefore sufficient to preserve gut homeostasis and intestinal immunity in a manner that is typically served by microbiota. With potential for such an influential impact, it should be no surprise that alterations and dysbiosis in the viral composition have been associated with several diseases and can alter host immune homeostasis, particularly within mucosal environments (142, 144).

### Virus-Mediated Regulation of Bacterial Communities

Using metagenomic analyses, researchers have observed the intestinal virome dramatically shifting prior to onset of T1D (23, 132, 145). Zhao et al., for instance, found that healthy donors had significantly higher viral diversity and increased abundance of *Circoviridae*-related sequences when compared to children who developed autoantibodies and T1D (132). These differences were observed prior to seroconversion and were also reflected in coinciding dysbiosis in bacterial communities. This suggests that there is a viral-bacterial relationship in precipitating autoimmunity. While modulation of commensal bacteria through phage bactericidal predation is not well understood, the ability of certain phages to affect bacterial abundance and modify bacterial fitness is particularly exemplified by the success of phage therapies in treating antibiotic-resistant bacterial infections (146). A study by Hsu et al. showed how phage-mediated killing has cascading effects within the microbiome, resulting in expansion or attrition of non-target bacterial populations and causing altered gut metabolomic profiles (147). These results suggest that lytic bacteriophages and the induction of prophages can be potent modulators of the bacterial microbiome and their effects can be amplified between molecular and cellular signals in the GI environment.

### Immune Regulation by Commensal Viruses

An exhaustive study by Dallari et al. characterized host immune responses to several asymptomatic virus infections (acute and persistent strains of MNV, mastadenoviruses, astrovirus, parvoviruses, and reoviruses) in conventional and germ-free mice (144). The authors identified both distinct and common immune modulation contributed by viral and bacterial microbes. Viruses were generally responsible for eliciting Th1- and IL-22-mediated immunity as well as B cell and bacterial response pathway activation. While each virus exposure promoted profound immunomodulation, there was little consistency in immune pathways activated by each virus examined. Viral genome type, virus persistence, and viral load were only modestly attributed to the observed immune variance suggesting there is a largely individualistic and strain-specific contribution to intestinal immunity. While bacterial members of the microbiome have been the major focus of research in respect to their ability to shape mucosal immunity, this highlights importance and impact virus exposure also has within both GI-related and systemic immune homeostasis.

Despite eukaryotic cells not being a natural target for bacteriophages, their presence can alter host immune profiles. This is most often accomplished by bacteriophage stimulation of viral PRRs, including TLRs or RIG-I-like receptors. One study showed how phage taken up in antigen-presenting cells activates TLR3 signalling and subsequently type I IFN expression (148). Another study demonstrated *Lactobacillus*, *Bacteroides*, and *Escherichia* phages can promote IFN-γ-producing T cells along with IL-6, IL-10, and IL-12 secretion *via* TLR9 activation in germ-free mice (149). These changes can alter susceptibility to ensuing bacterial and viral infection. For instance, the presence of murine astrovirus has been shown to protect against MNV and rotavirus infection *via* stimulation of type III interferon signalling in the gut epithelium (150). Type III IFN expression in epithelial cells may also be detrimental in determining persistence of CVB in enteric environments (151). Phage-mediated cell lysis of bacteria would also result in increased release of antigenic bacterial PAMPS that go on to initiate inflammation through PRR activation. Bacteriophage induced amyloid production in *E. coli* has been associated with subsequent seroconversion and development of T1D (152). This effect is hypothesized to be caused by the release of *E. coli* amyloid-DNA PAMPs, which are known inducers of TLR2 and TLR9 and have been previously shown to trigger SLE autoimmunity in mice (153). However, more evidence is needed to determine if this mechanism can be directly contributing to T1D.

### Human Endogenous Retroviruses

Ancestral viruses have integrated into the mammalian genome over millions of years of evolution, resulting in human endogenous retroviruses (HERVs). Some estimates attribute approximately 8% of the human genome to a viral origin (154). These genomic viral remnants largely go unexpressed. However, they can be induced by exogenous stressors including CVB and other viral infections (155–157). Expression of HERV antigen, particularly from the HERV-W family, has been associated with both T1D and multiple sclerosis autoimmunity in humans and mouse models (158–161). Mycobacterial infection can stimulate expression of the HERV-W envelope antigen, resulting in increased cross-reactive autoantibody expression in children at higher risk of T1D (162). Murine ERV antigens can be detected in the islets of NOD mice as disease progresses and anti-ERV immunity correlates with anti-islet reactivity (158). Furthermore, inducing expression of HERV-W-Env protein in mice causes hyperglycemia, reduced insulin production, and increased immune infiltration into the pancreas (159). This indicates a potential role in promoting inflammatory events within the islet microenvironment. While the exact role is to be determined, HERV-W-Env involvement in autoimmunity has been at least partially attributed to its signalling *via* CD14 and TLR4 PRR stimulation in APCs resulting in activation of Th1 and antimicrobial immune pathways (163).

### Molecular Mimicry in the Virome

Antigenic similarity between viral and host proteins can also potentially contribute to autoimmune responses. Antibodies against CVB4 viral protein can positively recognize beta cell antigen and induce cell apoptosis (164). Commensal viruses including Poxviruses, HHVs and other dsDNA viruses have been shown to exhibit sequence homology with multiple human peptide hormones such as insulin, insulin-like growth factors (IGFs), adiponectin, and resistin (165). Viruses in the *Iridoviridae* family express viral insulin/insulin-like growth-1-like peptides (VILPs), which share a significant homology with human insulin/IGF-1. These VILPS are able to adequately bind to, and cause activation of, their respective hormone receptors in both humans and mice (165). Whether this similarity can contribute to antigenic cross-reactivity against endogenous insulin in T1D has yet to be seen.

## Intestinal Commensal Bacteria Can Influence Virus Outcomes

There is a significant degree of bidirectional influence between the microbiome and antiviral response. Not only does infection alter the microbial homeostasis, but the microbiome can also have a significant impact on the outcome of virus infection and the ensuing immunological responses (144, 166–168). The microbiome has been shown to determine severity of viral infection and promote resistance to enteric infection (169–171). Certain species of commensal bacteria can colonize intestinal lymphoid tissues including the Peyer’s patches and mLNs to modify antigen-presenting cell cytokine expression even under healthy homeostatic conditions (172). There is some evidence that microbial antigens may even share sufficient homology to induce cross-reactive T cells against pancreatic targets (76, 122). For instance, an integrase protein expressed by many bacteria within the *Bacteroides* genus is capable of serving as a low-avidity mimotope of pancreatic autoantigen (173).

Commensal bacteria can aid or limit virus infection through enhanced viral genetic recombination, stabilization of virus particles, promotion of virion dissemination to permissive cells, and modification to immune homeostasis (174). Surface bacterial polysaccharides, such as peptidoglycan and LPS, have been shown to promote virion stability and receptor engagement to increase poliovirus and reovirus infectivity in mice (170). Certain *Bifidobacteria* and *Lactobacillus* species have even exhibited an inhibitory potential of CVB4 *in vitro* (175, 176). Additionally, depleting microbiota through use of antibiotics is able to reduce rotavirus infection by promoting virus-specific humoral responses (177). Infection with H3N2 and H1N1 influenza strains in mice causes intestinal dysbiosis and results in reduced SCFA production and diminished immune responses to secondary infections (135). Conversely, commensal bacteria LPS and extracellular matrix-binding proteins have also been shown to destabilize influenza virions and block infection at mucosal sites, respectively (178, 179).

### Microbial Activation of Antiviral Immunity

Intestinal bacteria can elicit prolonged steady-state activation of the innate and adaptive immune system to modify susceptibility to subsequent infection (180–182). For instance, commensal microbes can limit persistence of MNV infection in mice through stimulation of interferon signalling (183). Ultimately, bacterial stimulation of immune pathways may play an important role in setting the thermostat for ensuing pathogenic infections particularly in the intestinal environment (184). Antibiotic-treated mice have compromised innate and adaptive antiviral immune responses resulting in impaired ability to clear virus infection (181). This is likely because the sustained immunological stimulation from commensal microbiota lowers the activation threshold in order to establish a robust immune response against an invading pathogen. In fact, intestinal bacteria can send signals to lung stromal cells to maintain a primed baseline IFN signature to prepare against subsequent influenza infection and limit early viral replication (185). The antiviral thermostat may be altered in some individuals due to genetic variance and/or environmental stimulation. This may allow the establishment of persistent infections which have been observed prior to disease onset in individuals with islet-autoimmunity and T1D (23).

## Conclusion

Understanding how enteric viruses contribute to homeostatic regulation of immunity and may contribute to autoimmune disorders is of great importance. Consequences of virus exposure within the intestinal environment are difficult to determine due to a lack of established animal models and confounding variables including commensal microbes commonly found in murine colonies (e.g., SFB, astrovirus), which may limit viral infection and skew results (150, 186). Ultimately, mice also exhibit differences in viral susceptibility, tropism, and pathogenesis when compared to humans.

Changes in the microbiota have been observed to occur prior to autoimmunity development, which suggests that dysbiosis has a causative role in T1D rather than a result of autoreactive or metabolic pathophysiological responses (58). Intervention studies in humans modulating the microbiome through dietary means or FMT have shown some success in improving T1D outcomes and prevention (187). However, conclusive results in these studies may be limited and require further work.

While there remains much controversy with regards to the precise role and importance of virus infection and the microbiome in determining whether a genetically susceptible individual will lose self-tolerance, significant efforts are being made to understand the patterns and commonalities that are able break through the heterogeneity of human data, background noise, and experimental limitations currently impeding understanding of these issues. Mouse models certainly provide a great deal of potential mechanistic insight into T1D; however, longitudinal human studies integrating clinical data for microbiome differences, infection history, and susceptibility to T1D-related autoimmunity are absolutely necessary to dissect the complicated etiology leading to diabetes development. Blood and stool samples from these large cohort studies can shed light on changes in the microbial, viral, and immunological landscape prior to disease onset. Furthermore, intestinal inflammation and potential increases in gut permeability can be identified by determining abundance of blood markers, clinical tests, and presence of translocated bacterial antigen (188).

Communication between the intestinal microbiota and resident immune populations likely have a profound role in dictating susceptibility and immune system response to virus infection. The intimate inter-relatedness of genetic susceptibility, viral responses, dysbiosis, and host immune state produces an incredibly complex web whereby perturbation can cause a myriad of effects. Understanding the experimental complexity in host-virus-microbe interactions is a monumental challenge. It is difficult to determine which factors and pathways are active contributors to, rather than incidental by-products of, disease. Though challenging, exploring this relationship further is necessary to inform the ultimate prevention, detection, and treatment of autoimmunity.

## Author Contributions

ZM and MH conceptualized, wrote, and edited the manuscript. ZM created the figures. All authors contributed to the article and approved the submitted version.

## Funding

The work was funded by grants from the Juvenile Diabetes Research Foundation and GenomeBC.

## Conflict of Interest

The authors declare that the research was conducted in the absence of any commercial or financial relationships that could be construed as a potential conflict of interest.

## Publisher’s Note

All claims expressed in this article are solely those of the authors and do not necessarily represent those of their affiliated organizations, or those of the publisher, the editors and the reviewers. Any product that may be evaluated in this article, or claim that may be made by its manufacturer, is not guaranteed or endorsed by the publisher.

## Abbreviations

AMP, antimicrobial peptide; APC, antigen-presenting cell; CAR, coxsackie and adenovirus receptor; CARD, caspase activation and recruitment domains; CTLA4, cytotoxic T-lymphocyte associated protein 4; CVB, coxsackievirus B; dsRNA, double stranded RNA; DSS, dextran-sulfate sodium; EBV, Epstein-Barr virus; FMT, fecal microbiome transfer; GAD, glutamic acid decarboxylase; GI, gastrointestinal; HAdV-C, human masadenovirus-C; HERV, human endogenous retroviruses; HHV, human herpes viruses; HLA, Human leukocyte antigen; IEB, intestinal epithelial barrier; IFIH1, interferon induced with helicase C domain 1; IFN, interferon; IGF, insulin-like growth factors; IL2RA, interleukin-2 Receptor alpha; LPS, lipopolysaccharide; MAIT, Mucosa-associated invariant T cells; MHC, major histocompatibility complex; mLN, mesenteric lymph node; MNV, murine norovirus; MR1, MHC class 1-related protein; MyD88, Myeloid differentiation primary response 88; NOD, non-obese diabetic; NOD2, nucleotide-binding oligomerization domain-containing protein 2; NOR, non-obese diabetes resistant; PAMP, pathogen-associated molecular patterns; pDC, plasmacytoid dendritic cells; PRR, pattern recognition receptor; PTPN22, protein tyrosine phosphatase 22; SCFA, short chain fatty acid; SLE, systemic lupus erythematosus; T1D, type 1 diabetes; Th, T helper cell; TLR, toll-like receptor; Treg, regulatory T cell; VILP, viral insulin/insulin-like growth-1-like peptides.
